# Correlation of Sociodemographic Factors, Characteristics of Burns, and Neutrophil-to-Lymphocyte Ratio with the Level of Depression in Patients with Burn injuries

**DOI:** 10.1016/j.jpra.2024.04.005

**Published:** 2024-04-12

**Authors:** Hardisiswo Soedjana, Lisa Y. Hasibuan, Betha Egih Riestiano, Santi Andayani, Annisa Nurfitriani, Ali Sundoro, Almahitta Cintami Putri, Arif Tri Prasetyo

**Affiliations:** a Division of Plastic Reconstructive and Aesthetic Surgery, Department of Surgery, Faculty of Medicine Universitas Padjadjaran, Dr. Hasan Sadikin General Hospital, Bandung, West Java, Indonesia; b Department of Psychiatry, Faculty of Medicine Universitas Padjadjaran, Dr. Hasan Sadikin General Hospital, Bandung, West Java, Indonesia

**Keywords:** Depression, Burns, Neutrophil-to-lymphocyte ratio (NLR)

## Abstract

**Background:**

The most common psychological impact experienced by patients with burn injuries is depression. Several significant risk factors influence depression, including sociodemographic factors (gender, employment status, socioeconomic status, and marital status) and burn characteristics (burn depth, burn area, and total burn surface area). Neutrophil-to-lymphocyte ratio (NLR) was discovered as a new biomarker for depression detection. The purpose of this study was to investigate the correlation of sociodemographic factors, burn characteristics, and NLR with the severity of depression in patients with burn injuries.

**Methods:**

This analytic descriptive study was conducted at Dr. Hasan Sadikin General Hospital from June 2022 to April 2023. Samples were assessed by a psychiatrist using the Hamilton Depression Rating Scale instrument, and a differential white blood count was calculated to obtain the NLR value.

**Results:**

The study sample consisted of 32 patients, including 27 males and 6 females. There was no correlation of sociodemographic factors and burn wound characteristics with the level of depression. NLR in patients with burn injuries who had no depression, mild depression, and moderate depression was 5.78 ± 2.23, 7.4 ± 1.77, and 13.04 ± 6.25, respectively. NLR was statistically significantly associated with the level of depression in patients with burn injuries (p = 0.001).

**Conclusion:**

There was no correlation of sociodemographic factors and burn characteristics with the level of depression. There was a significant correlation between NLR and the level of depression in patients with burn injuries at Dr. Hasan Sadikin General Hospital.

## Background

Burn cases pose a global public health challenge, serving as a primary cause of substantial illness and mortality rates in developing nations. The majority of global incidents arise in African and Southeast Asian countries, contributing to an annual mortality rate of 180,000 worldwide.^1^ As per the 2018 Riskesdas national report, Indonesia experiences a burn prevalence of 1.3% (1,200 cases annually) among a total of 92,976 patients with trauma. The medical records from Dr. Hasan Sadikin General Hospital for 2015-2017 indicated 373 recorded burn cases.^2^

Burn injuries necessitate extended hospitalization and ongoing treatment, leading to a range of physical and psychological issues. Although advancements in medical care have increased survival rates during the acute phase, a novel challenge emerges in the form of enduring psychological effects, particularly depression, which afflicts approximately 42% of patients with burn injuries.^3^, ^4^, ^5^, ^6^

Factors significantly contributing to postburn depression include preburn individual factors (e.g., gender, pre-existing psychiatric history, socioeconomic status, and marital status), burn-specific aspects (e.g., burn depth, area, and severity), and factors aiding resilience during recovery (e.g., social support and coping mechanisms). Notably, these correlations or predictors of depression vary across different studies.^7^^,^^8^

A noteworthy research at Kilpauk Medical College Government Hospital, India, found that depression was prevalent in 50% of cases within 1 month of treatment and 26% of cases within 3 months after burn.^9^ In the Burns Unit Cipto Mangunkusumo General Hospital, Jakarta, during April-May 2017, 57.1% of patients exhibited psychopathology, with 69.6% exhibiting signs of depression. Depression's impact encompasses cognitive, emotional, motivational, social, and physiological disruptions in the recovery of patients with burn injuries.

Depression increases the risk of medical complications and mortality because of poor patient compliance.^10^ It is also correlated with increased pain and reduced physical function among patients with burn injuries.^11^^,^^12^ Depression's effects on appetite and nutrient intake further impede wound healing. Patients with depression may have lower levels of serum albumin, as noted by Huang et al.^13^

Recent studies have underscored the influence of depressive states on the immune system, which elevate the risk of infection.^4^^,^^14^ Inflammatory mediators, particularly neutrophils and leukocytes, are crucial in this context.^15^, ^16^, ^17^ The neutrophil-to-lymphocyte ratio (NLR) emerges as a significant inflammation marker, with higher NLRs associated with increased incidence of major depression.^17^

In 2013, the American Burn Association convened to establish guidelines for psychiatric screening and diagnostic tools for burn survivors.^6^ The Hamilton Depression Rating Scale (HDRS) is commonly used for assessing depression levels. Inflammatory biomarkers, including C-reactive protein (CRP), interleukins, and NLR, have been explored in relation to depression.^5^^,^^18^^,^^19^ Measurements using HDRS tools are performed by psychiatric doctors who have attended standardization training and currently have a validated Indonesian version of HDRS. Research using HDRS tools has also been conducted in previous studies on patients with burn injuries in India.^5^

A retrospective study of patients with extensive burn injuries at Changhai Hospital demonstrated that NLR values decreased after treatment, suggesting NLR's potential as a survival predictor. Despite numerous studies on patients with depression and burn injuries, no research has specifically addressed the connection between NLR and depression in this context, both internationally and in Indonesia.^20^

Neutrophils and leukocytes play an important role in the inflammatory process. Neutrophils are the most abundant type of white blood cells (WBCs). Neutrophils and leukocytes play an important role in the course of inflammatory diseases. They also play an important role in the inflammatory response. Neutrophils are the first cells to respond to inflammation, especially that caused by bacterial infections, cancer, and environmental exposure. Neutrophils cause the secretion of several inflammatory cytokines.^15^

NLR can be derived from WBC. Cell counting is a cheap and reproducible test and has been investigated as a novel biomarker for systemic inflammatory response.^21^ NLR has emerged as an important inflammatory marker, and high NLR levels are associated with an increased incidence of major depression.^22^^,^^23^

Inflammatory processes in the brain resulting in an imbalance of inflammatory mediators can affect neurotransmitter metabolism (glutamate and serotonin), which has an impact on neuropsychiatric disorders such as depression.^24^^,^^25^

Many studies have shown that depression is associated with interactions among the central nervous system, immune response, and vascular reactivity. The immune system is altered during depressive conditions, which can suppress the immune system. Previous studies have shown that depression is associated with increases in inflammatory markers, including CRP, interleukin (IL)-6, and IL-1.^26^^,^^27^ Although inflammatory cytokines are useful biomarkers, the cost of testing is quite high, and testing facilities are still limited.

Understanding the extent of depression and its correlation with NLR in patients with burn injuries remains a critical research gap.^4^ Sunbul et al.^15^ evaluated depressive disorders using HDRS and NLR in patients at the Psychiatric Polyclinic, Istanbul, Turkey, in 2013. Based on the results of their study, NLR > 1.57 was a significant independent predictor of severe or very severe depression in patients (p = 0.034).^28^ Such knowledge could greatly aid in screening, early detection, and personalized psychological interventions, promoting recovery of patients with burn injuries. The convenience of NLR assessments could facilitate integrated treatment within Dr. Hasan Sadikin General Hospital inpatient facilities.

## Methods

This prospective study was performed at Dr. Hasan Sadikin General Hospital, Bandung, Indonesia. This research aimed to study the correlation of risk factors and effects with approach, observation, or data collection techniques at a certain time (point time approach). Judging from the relationship between the variables, this is a correlation research, i.e., research to explain the relationship between variables in which 1 variable causes or determines the value of another. The research sample was taken by consecutive sampling. The subject population included patients with burn injuries. The target population of this study was all patients with burn injuries. The reachable population was patients with burn injuries hospitalized at Dr. Hasan Sadikin General Hospital from June 2022 to April 2023 or when the sample was fulfilled. Samples were taken from populations that met the inclusion and exclusion criteria.

Inclusion criteria for this study were patients with burn injuries with indications for hospitalization according to Australian and New Zealand Burn Association guidelines, those with onset of burn events <24 hours before being taken to the hospital, those aged 18-60 years, and those who agreed to participate in the study by signing an informed consent sheet. Exclusion criteria for this study were pregnant and lactating women; patients with a history of autoimmune diseases, hematological disorders, malignancy, acute infections, chronic inflammation, 3 months of glucocorticoid consumption, and chronic kidney and liver function disorders; and patients with a history of mental disorders who were screened by the psychiatric team using the Depression Anxiety Stress Scale (DASS) tools.^29^

The dropout criteria for the study were patients with coronavirus disease 2019 who died/went home before 2 weeks of treatment, patients with decreased consciousness with Glasgow Coma Scale score of <15, or patients intubated in care for 14 days (diagnostic criteria for depression based on *Diagnostic and Statistical Manual of Mental Disorders, Fifth Edition*). The minimum number of patients included in this study was 30 based on calculation of the population because there was no previous similar correlation study.

Steps for collecting data were as follows:

• Patients with burn injuries treated at Dr. Hasan Sadikin General Hospital who met the inclusion criteria were also offered to participate in the study, and the patients who agreed to write on the informed consent sheet that had been approved by the Dr. Hasan Sadikin General Hospital Ethics Committee were included.
• Patients were observed for 14 days of treatment, and those who survived until the 14th day of treatment underwent hematology blood tests with a type count to be analyzed by the Laboratory of Dr. Hasan Sadikin General Hospital.
• Patients were screened on the 14th day of treatment and interviewed using the HDRS by 2 permanent residents who conducted the study.
• Results of screening interviews using the HDRS Indonesian version were calculated, and the scores were totaled by 2 psychiatric residents in consultation with a psychiatric specialist doctor (all the examiners were trained to conduct the interview with validated Indonesian version of HDRS).
• Data were collected regarding the results of the assessment and analyzed after they had been collected; then, the results of the research were reported at the end.

The data obtained were recorded in a form and then processed through SPSS version 23.0 for Windows. The significance criterion used was the p-value; p-value of ≤0.05 was considered statistically significant or significant.

## Results

All study patients were assessed for depression using the HDRS; then, the scores were calculated and classified into mild, moderate, severe, and very severe depression. Patients with HDRS scores of 0 to 7 were considered to have no depression), those with scores 8 to 13 were considered to have mild depression, those with scores 14 to 18 were considered to have moderate depression, and those with scores 19 to 22 were considered to have severe depression.

In this study, the majority of burn victims experienced burns with an area of >20% of the total body surface area (TBSA). Table 1 shows the condition of patients with burn areas of >20% of the TBSA, and 66.7% of patients experienced mild depression. In addition, each patient was assessed for the deepest burn experienced. From this assessment, 15 patients had full-thickness burns, 11 had deep dermal burns, 4 had mid-dermal depth burns, and 2 had superficial burns. Based on the burn area, 21 (65%) patients experienced burns on the face area, accompanied by burns on other areas such as the upper extremities, lower extremities, trunk, or genitals.

**Table 1:** Characteristics of patients.

Variables	No depression (n = 11)	Mild depression (n = 17)	Moderate depression (n = 4)
Depth of wound			
Superficial dermal (n = 2)	1 (50%)	0 (0%)	1 (50%)
Mid-dermal (n = 4)	3 (75%)	1 (25%)	0 (0%)
Deep dermal (n = 11)	4 (36.4%)	5 (45.5%)	2 (18.2%)
Full thickness (n = 15)	3 (20%)	11 (73.3%)	1 (6.7%)
Wound area			
Extremities (n = 6)	2 (33.3%)	3 (50%)	1 (16.7%)
Face, extremities (n = 6)	3 (50%)	3 (50%)	0 (0%)
Body, extremities (n = 5)	2 (40%)	3 (60%)	0 (0%)
Face, body, extremities (n = 12)	3 (25%)	7 (58.3%)	2 (16.7%)
Face, body, extremities, genital (n = 3)	1 (33.3%)	1 (33.3%)	1 (33.3%)
TBSA			
<20% (n = 11)	6 (54.5%)	3 (27.3%)	2 (18.2%)
>20% (n = 21)	5 (23.8%)	14 (66.7%)	2 (9.5%)

Table 2 shows the analysis conducted between sociodemographic factors, such as gender, occupation, income, and marital status, and depression levels. The results of Pearson correlation test obtained from the results of data analysis revealed that the factors gender, income, and marital status were not significantly correlated with the level of depression in patients with burn injuries.

**Table 2:** Correlation of variables with depression.

Variables	*P*
Correlation of sociodemographic status with depression	
Gender	0.713
Monthly income	0.373
Marriage status	0.378
Correlation of burn characteristics with depression	
Wound area	0.875
Wide area	0.315
Depth of wound	0.567

**Figure 1:**
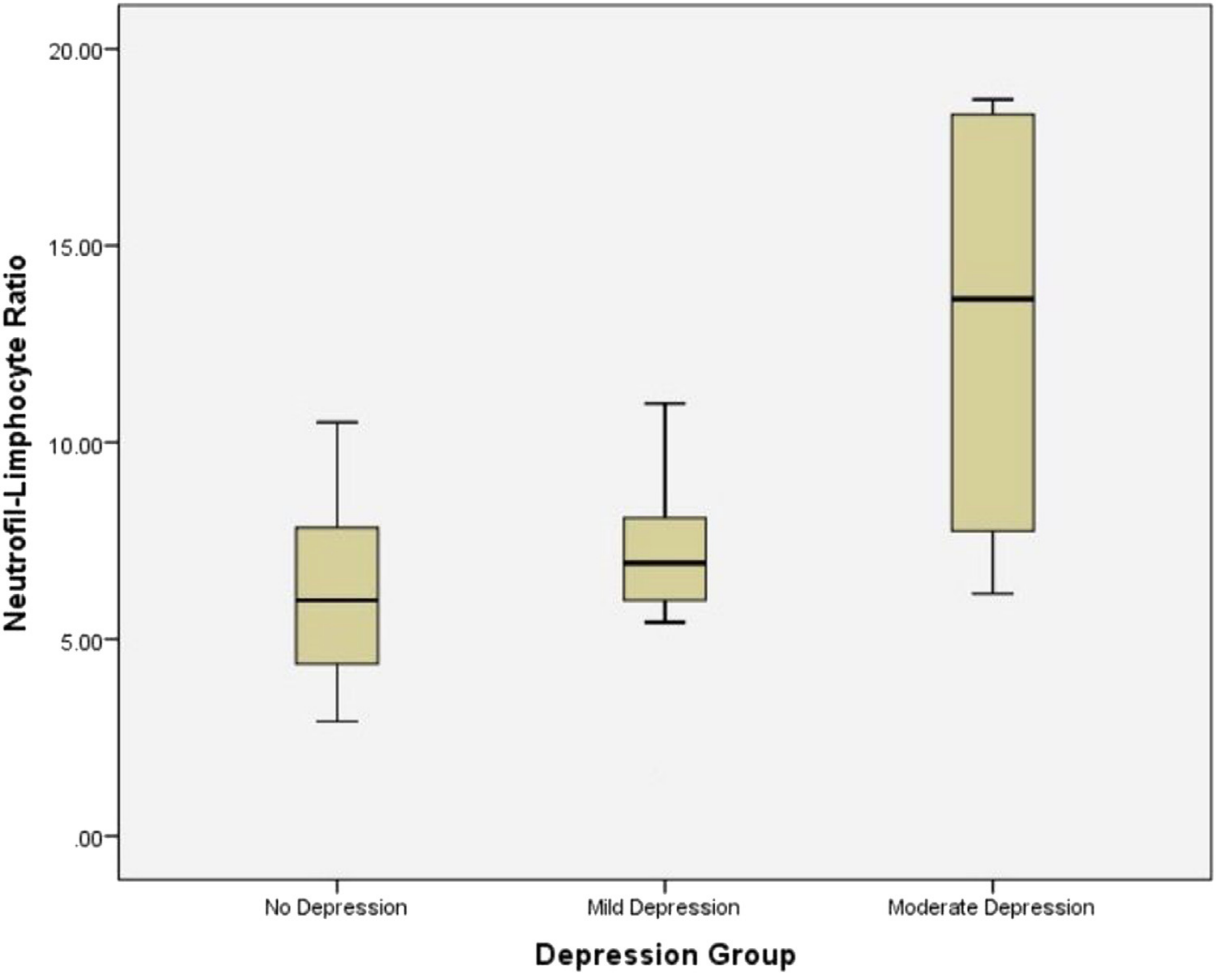
Comparison of neutrophil-to-lymphocyte ratio of patients with burn injuries between depression groups.

Most patients experienced depressive conditions ranging from mild to moderate, and none had severe depression. Among those with full-thickness burns, 73.3% had mild depression compared with those with deep dermal burns, and only 45.5% had mild depression. The close relationship between NLR and the level of depression in patients with burn injuries on the 14th day was determined by the Pearson correlation test. It was found that patients with higher levels of depression had a much higher level of NLR than patients with lower levels of depression (Figure 1). Most patients were treated in semi-intensive rooms (91%), and the length of hospital stay ranged from 14 to 28 days (68%). Half of the patients had no other accompanying complications in burn wound care, and some had compartment syndrome (15%), transaminitis (15%), and bronchopneumonia (3%). All study patients did not have accompanying inhalation trauma because intubated patients were included in the exclusion criteria.

NLR ± standard deviation was 5.78 ± 2.23 in patients who were not depressed, 7.4 ± 1.77 in those who were mildly depression, and 13.04 ± 6.25 in those who were moderately depressed. Correlation analysis showed that the level of depression was significantly related to NLR (p = 0.001) and neutrophils (p = 0.032) in patients with depressive disorders (Table 3). As shown in Table 4, statistical analysis by the Pearson correlation test was conducted between NLR and depression levels in patients with burn injuries (p = 0.001; p < 0.05). There was a significant correlation between neutrophil yield and the depression level of patients with burn injuries (p = 0.032; p < 0.05).

**Table 3:** Comparison of neutrophils, lymphocytes, and neutrophil-to-lymphocyte ratio.

	No depression (n = 11)	Mild depression (n = 17)	Moderate depression (n = 4)	p
Neutrophils (1/mm^3^)	10.34 ± 2.88	12.07 ± 3.97	19.19 ± 7.64	0.032
Lymphocytes (1/mm^3^)	1.94 ± 0.66	1.63 ± 0.45	1.49 ± 0.3	0.069
NLR**	5.78 ± 2.23	7.4 ± 1.77	13.04 ± 6.25	0.001

p < 0.05. **statistically significant.

**Table 4:** Correlation of neutrophils, lymphocytes, and neutrophil-to-lymphocyte ratio with depression.

Variable	Correlation	ρ	p
Neutrophils with depression	Spearman	0.331	0.032**
Lymphocytes with depression	Spearman	−0.268	0.096
NLR with depression	Spearman	0.519	0.001**

p < 0.05. **statistically significant. ρ, coefficient correlation.

Table 4 shows the correlation coefficients of neutrophils, lymphocytes, and NLR on the level of depression in patients with burn injuries. The correlation coefficient of NLR (ρ = 0.519) showed that the direction of the positive correlation with a strong correlation strength with the level of depression in patients with burn injuries was moderately correlated, whereas the correlation coefficient of neutrophils (ρ = 0.331) showed a small/not tight correlation strength.

## Discussion

This analytical investigation aimed to enhance our comprehension of depression in patients with burn injuries by assessing depression levels at the 2-week mark of burn care. Sociodemographic baseline information and burn-related patient characteristics were integrated into the analysis to explore how demographic factors may influence depression. A deeper understanding of these demographic predictors and burn characteristics could lead to improved detection, treatment, and even prevention of depression in this specific population.

In alignment with many other studies, our observations uncovered a sense of depression among patients with burn injuries, but only a minority of the patients exhibited symptoms of major depression.^6^ Among hospitalized patients with burn injuries, self-reported levels of depression ranged from moderate to severe (10%-54%).^4^^,^^9^^,^^12^^,^^30^, ^31^, ^32^ In studies involving adults with severe burns, the prevalence of major depression during hospitalization was approximately 4%, with clinically significant depressive symptoms varying from 8% to 35%. Differences in prevalence might arise from diverse assessment tools, timing, sample size, or burn severity issues.^33^ Variances in study outcomes may also underline the challenges in evaluating depressive symptoms within the population of patients with burn injuries. It is worth noting that physical burn-related symptoms may considerably overlap with somatic symptoms used to diagnose depression, creating complexities in evaluation.^7^^,^^34^

A significant finding from our research is that although major depression rates during hospitalization were low, a notable proportion of patients experienced mild-to-moderate depression. This could partly be attributed to patients with pre-existing depression being excluded from the sample.^6^ Although adults may express anxiety through “panic” symptoms such as sweating, palpitations, trembling, or nausea, children may express anxiety by crying, throwing tantrums, freezing, or clinging. A differential diagnosis of these anxiety disorders distinct from post-traumatic stress disorder is difficult and requires a careful interview.

Ren et al.^28^ explored the correlation between burn injuries and the onset of delirium, focusing on identifying risk factors that could lead to delirium in patients with burn injuries, emphasizing the critical need for early detection and intervention strategies to mitigate these risks, thereby enhancing patient recovery and outcomes in burn care settings. The review serves as a crucial resource for healthcare professionals, providing insights into the mechanisms of delirium and suggesting avenues for future research and clinical practice improvements.^28^

Follow-up of NLR levels was performed on day 14. According to the diagnostic criteria for the onset of depression and in accordance with the course of inflammation, NLR levels will reach their highest value on days 7-28. NLR at 24 hours rises, and on day 3, it returns to normal, and then, it will rise again until 7th day and remain at high levels until 28th day.^35^

Additionally, the burn unit's rigorous pain management approach at Dr. Hasan Sadikin General Hospital could play a role in lowering depression rates, considering the substantial pain, itching, and sleep disturbances inherent in burn injuries and treatments.

Our study's results on sociodemographic characteristics of patients with burn injuries revealed that a majority of the patients were married, a factor that can contribute to psychosocial stress due to increased familial responsibilities. Additionally, a significant proportion of patients had low socioeconomic income, aligning with global trends, where most burns occur in lower socioeconomic settings. Prior history of depression and lower well-being were stronger predictors of depression than burn size. This is consistent with previous findings suggesting that pre-existing depression or lower well-being are more influential in precipitating depression than the extent of burn injury.^6^

Our study found that factors such as burn area, burn depth, and extent of burn do not correlate with depression, which is in line with prior research.^4^^,^^5^ Notably, our study revealed that NLR results correlated with depression severity. This observation aligns with previous research, indicating that NLR could serve as a simple and cost-effective method to assess depression severity, especially in the absence of conditions that might trigger inflammatory responses. Incorporating WBC count assessment into psychiatric evaluations of patients with burn injuries could aid in identifying those at risk of depression.^4^

Pathophysiological events result in breakdown of the blood-brain barrier and development of cerebral edema after burn injury. After severe burn trauma, a strong systemic inflammatory response is triggered. Proinflammatory mediators are produced by various immune cells, resulting in breakdown of the blood-brain barrier, followed by activation of central nervous system cells, such as microglia and astrocytes, which respond with further production of inflammatory markers, accumulating in a massive neuroinflammatory response, and life-threatening cerebral edema. In parallel, significant hormonal changes are triggered, resulting in a severe hypermetabolic state.^36^

Psychiatric morbidity, especially depression, is common in adults because of burns, trauma, and other critical illness survivors. In addition to adverse mental health outcomes, these patients are also at risk of impaired quality of life and reduced ability to return to work.^33^ Future studies are needed to develop a greater understanding of risk factors for psychopathology and further preventive interventions and/or treatment for psychiatric disorders in burn, trauma, and intensive care unit survivors.

This study offers valuable clinical insights, highlighting the association between NLR and depression severity in patients with burn injuries. The simplicity and affordability of NLR assessment make it a potential tool for gauging depression severity, both in inpatient and outpatient settings. Integrating NLR assessment could guide follow-up strategies for patients with burn injuries exhibiting higher NLR rates, suggesting a tendency toward depression.

There are several limitations to this study. First, the study did not use structured clinical interviews to diagnose depression, although HDRS has been used widely to measure depression levels and has been shown to have a high correlation with formal diagnostic interviews.^6^^,^^18^^,^^19^ Assessment of psychiatric comorbidities is performed only once; therefore, if there are psychiatric symptoms that occur later, they are not recorded because there is no follow-up. We know that a history of psychiatric disorders puts people at increased risk of burn injuries and depression. Therefore, it is likely that the level of depression will be higher if these patients participating in this study are anticipated by assessing the DASS. DASS is a reliable measurement with a validated Indonesian language format and is used to measure negative emotional states of depression, anxiety, and stress.^29^ However, in this study, the DASS assessment was conducted simultaneously with the HDRS assessment; hence, an assessment of the history of psychiatric disorders on day 14 could be biased.

In addition, this study focused only on assessing the level of depression. Future studies are warranted to extend the length of time these patients are followed up to check the level of depression after hospitalization. Various personal, social, and environmental factors that may contribute to psychological problems in patients have not been explored in this study.

There are still important questions about depression in the burn population, which future researchers may wish to consider. Given that pain levels during hospitalization are closely related to depression, future research may be able to focus on the relationship between depression and pain in the inpatient setting. In the chronic pain literature, approximately 33% of patients meet criteria for depression, and another 33% exhibit a large number of depressive symptoms. Many patients with burn injuries experience pain, either neuropathic or muscular, upon discharge, which can have a significant impact on mood and adjustment.^6^

Burn units worldwide recognize the importance of the biopsychosocial model in the care of patients with burn injuries and generally function as a multidisciplinary team to meet the diverse needs of these patients, both during hospitalization and after discharge. The ability to identify and respond to patient psychiatric disorders is an integral part of the recovery and rehabilitation of patients with burn injuries. This study provides further evidence that depression is 1 of the most common psychiatric disorders and may be the most detrimental to recovery. These data indicate that there is a significant pressure on patients with burn injuries who can immediately receive intervention starting with early detection. Early detection, followed by appropriate intervention, will encourage the patient's physical recovery. All patients with burn injuries must be routinely screened for psychiatric morbidity at least once during their hospitalization.

This study has important clinical implications. To the best of our knowledge, we have demonstrated for the first time the association between NLR and depression severity in patients with burn injuries. We show that the level of NLR may be related to the severity of depression in patients who definitely do not have other conditions that can activate the inflammatory response. NLR seems to be a simple, cost-effective method for evaluating the severity of depression in depressive disorders and can be used in inpatient or outpatient care. Determining the WBC count is simple and affordable and can provide an overview of the severity of depression. It should be included in the psychiatric evaluation of patients with burn injuries so that follow-up can be performed for patients with burn injuries who have higher rates of NLR with a tendency for depression.

## Conclusion

There was no correlation between sociodemographic characteristics and the level of depression in patients with burn injuries. However, there was a significant correlation between neutrophils and NLR and the level of depression in patients with burn injuries.
